# Prevalence and predictive factors for infertility-related stress among infertile couples: A cross-sectional study from Jordan and the occupied Palestinian territories

**DOI:** 10.15537/smj.2022.43.10.20220411

**Published:** 2022-10

**Authors:** Deema J. Jaber, Haneen A. Basheer, Abla M. Albsoul-Younes, Lina M. Elsalem, Jehan M. Hamadneh, Mohammad K. Dweib, Hanadi T. Ahmedah

**Affiliations:** *From the Department of Clinical Pharmacy (Jaber, Basheer), Faculty of Pharmacy, Zarqa University, Zarqa, from the Department of Clinical Pharmacy (Albsoul-Younes), University of Jordan, Amman, from the Department of Pharmacology (Elsalem); from the Department of Obstetrics and Gynecology (Hamadneh), Faculty of Medicine, Jordan University of Science and Technology, Irbid, Jordan, from the Department of Clinical Pharmacy (Dweib), Faculty of Pharmacy, Hebron University, Hebron, Palestine, and from the Department of Medical Laboratory Technology (Ahmedah), Faculty of Applied Medical Sciences, King Abdulaziz University, Rabigh, Kingdom of Saudi Arabia.*

**Keywords:** depression, anxiety, stress, reproductive health, infertility, mental health

## Abstract

**Objectives:**

To assess the level of infertility-related stress, associated socio-economic, and demographic factors among infertile couples living in Jordan and those living under the chronic Israeli-Palestinian conflict in the occupied Palestinian territories.

**Methods:**

A cross-sectional study was carried out in a number of fertility and reproductive clinics in Jordan and occupied Palestinian territories over a period of 6 months. Trained clinical pharmacists interviewed the identified couples.

**Results:**

A total of 443 participants were interviewed. Three variables were significantly and independently associated with global stress scores. The need of parenthood appears higher in women than men among infertile couples in Jordan and Palestine (*p*=0.005). The country of origin (*p*<0.001) made the greatest contribution of unique variance followed by family type (*p*=0.035). Additionally, a significant contribution to the model was carried out by the number of clinicians who followed up on the case (*p*=0.013). The average total cost of treatment since the problem had been diagnosed was 2936±4529 Jordanian dinar, which may be of concern to both Jordanians and Palestinians given the limited resources available in developing nations.

**Conclusion:**

This study shows a significant degree of stress among infertile couples. The place of origin, family structure, and presence of medical insurance had a significant impact on the infertility global stress score. This study emphasizes the necessity for specific psychological therapies that are currently lacking in public healthcare practices in both Jordan and Palestine.

**T**he failure to become pregnant after a year of routine, unprotected sexual activity is known as infertility.^1^ Approximately 15% of married couples worldwide struggle with infertility, which prevents them from having children and causes them financial pressure and emotional pain.^2,3^ A global survey found that women were more likely than men to have infertility, which may be due to cultural differences in how much men are reported as experiencing infertility.^4^ In Jordan, primary infertility rate have been estimated to be 3.5% and secondary infertility rate have been estimated to be 13.5%.^5^ With a rate of approximately 15% in 2016 in the occupied Palestinian territories, infertility among couples is an issue, according to the World Health Organization (WHO).^6^

Parenthood is viewed by society and culture as a crucial component of marriage, and infertile couples face difficulties when they are unable to conceive.^5,7,8^ These difficulties can occasionally lead to infertility-related stress characterized by anxiety, depression, sadness, and feelings of worthlessness and isolation, all of which have a negative impact on both life quality and lifespan.^9-11^ Depression is the most prevalent mental health issue worldwide and a significant public health issue among these psychological diseases.^12^ One of the few options for women to improve their standing in their families and communities in Arab nations and patriarchal civilizations, such as Jordan and the occupied Palestinian territories, is through motherhood and having children.^13^ Infertility and the difficulties that Jordanian women face in their personal, marital, and social lives have a significant negative impact on their health.^14^

Alive births are achieved by approximately half of all infertile couples with the aid of assisted reproductive technology (ART), and this can improve the psychological consequences of being infertile.^15^ On the other hand, distress and anxiety can lower an infertile couple’s chance of success with ARTs such as in vitro fertilization (IVF).^16^ The use of antidepressants may also affect male and female fertility.^17,18^ Therefore, studies carried out to evaluate the psychological wellbeing of infertile couples are essential to develop a more accurate clinical care and successful treatment.

It is unclear how common infertility-related stress in poor countries like Jordan and Palestine due to a lack of registration and detailed research addressing infertility-related stress in the Arab world in general. Accordingly the main objective of this cross-sectional study is to evaluate the level of infertility-related stress, associated socio-economic, and demographic factors among infertile couples living in Jordan and those living under the chronic Israeli-Palestinian conflict in the occupied Palestinian territories.

## Methods

Between December 2018 and February 2020, a cross-sectional survey was carried out in Jordan and the occupied Palestinian territories. It involved couples who visited the fertility and reproductive clinics at Jordan University Hospital (JUH), Amman, Jordan, and King Abdullah University Hospital (KAUH), Rabigh, Saudi Arabia, and at 5 private fertility centres in the occupied Palestinian territories (Health Work Comittes, Hebron, Omar Medical Center, Bethlehem, Bait Almaqdes Medical Center; German Center; and Istanbul Medical Center, Jerusalem). We selected fertility and reproductive centres that are mostly visited by infertile couples.

Participants in the study voluntarily and anonymously provided their consent. Each pair gave their signed consent prior to data collection after being informed of the study’s goal, procedures, and potential benefits.

The inclusion criteria included: consenting infertile couples (failure to become pregnant after a year of unprotected sexual activity is the medical definition of infertility) with primary infertility. The exclusion criteria included: couples who had been treated for infertility and suffering from personally stressful life events (such as change of residence, work loss, loss of relatives, and being a victim of crime) in the last 6 months, suffering from mental health disorders, or receiving treatment.

There was convenience sampling. Participants were informed regarding the study and offered to take part in interviews while awaiting a doctor’s appointment or getting their medications filled at the pharmacy.

Based on the number of research couples necessary to do regression analysis, sample size was determined (5-20 study research subjects per predictor).^19^ Given that we have 20 predictors and a medium number of 10 study participants per predictor, a minimum sample size of 200 for each country (400 in total) seemed suitable.

The information was gathered using a standardized, verified questionnaire that was administered by an interviewer. The identified couples were interrogated by skilled clinical pharmacists. To reduce the possibility of responses being influenced, each participant was questioned separately from his or her partner.

The study adhered to the Declaration of Helsinki recommendations carried out by the World Medical Association. The study was approved by the Deanship of Research and the Institutional Review Boards committee of Zarqa University, Zarqa, Jordan, JUH, Amman, Jordan, and KAUH, Rabigh, Saudi Arabia, (IRB number 64/118/2018) to ensure ethical procedures in data collection and analysis.

The 3 sections of the questionnaire utilized in this study were as follows: I) the first part elicited the socio-demographic characteristics of participants in terms of gender, age, smoking, education level, occupation, and suffering from any chronic diseases; II) the second part collected data on marriage duration, family type (nuclear or extended; an extended family was defined as a family composition that includes near relatives and the nuclear family in one household, and where the relationships between the members are biological and social; it is a constellation of nuclear families across 2 or more generations),^20^ infertility duration, infertility treatment (duration, method, costs, funding sources, and coverage), number of gynaecologists involved in follow up to the case, family members suffering from infertility, and whether there was psychological consultation for infertility related to stress; III) the final part assessed infertility-related stress using the Arabic language version of the Fertility Problem Inventory (FPI) assessment tool, adapted from the original English version.^21,22^ In this 46-item scale, infertile couples were asked to respond on a 6-step Likert-scale to how much they agreed or disagreed with various attitudes or worries connected to infertility.

Composite FPI subscales (social concern, sexual concern, relationship concern, need for parenthood, and rejection of childfree lifestyle) were calculated individually. The scores of all 5 perceived infertility-related stress subscales were added to create the final FPI total scale (Global Stress). Patterns of infertility-related stress assessed by the 5 FPI subscales scales were found to differ by demographic and infertility-related characteristics. Scores of 92 or below were obtained to indicate low infertility-related stress, scores of 93-123 to indicate average infertility-related stress, scores of 124-157 to indicate moderately high infertility-related stress, and scores of 158 or more to indicate extremely high infertility-related stress.

High reliability, good discriminant validity, and convergent validity have all been proven for the FPI.^22^ Each item’s dimension was tested for reliability using Cronbach’s alpha, and the results were 0.86, indicating good reliability and construct validity.

The research team, made up of 5 academics and 2 gynecologists, reviewed each questionnaire utilized to ensure face validity. The research team carried out revisions and contrasted the translated Arabic questionnaire with the original English questionnaire. Prior to the official data gathering process beginning, the study was piloted with 10 volunteers. Few questions were modified as a result, and these data were not included in the final study.

### Statistical analysis

Data were entered, coded and analysed using the Statistical Package for the Social Sciences, version 22.0 (IBM Corp., Armonk, NY, USA). Frequencies/percentages and means/standard deviations (SD) were used to analyze qualitative and quantitative descriptive data. Independent T-tests and ANOVA were used to evaluate the association between different demographic variables and different infertility stress components.

Independent factors correlating with infertility-related stress were determined using simple linear regression. Subsequently, any variable with a *p*-value of <0.05 was entered into multiple regression analysis utilising backward elimination method. Statistical significance was defined as *p*<0.05. Checks for normality were carried out using the Shapiro-Wilk test (with a *p*-value of ≥0.05 that indicates a normally distributed continuous variable). Before carrying out the multiple linear regression analysis, it was made sure that there was no multicollinearity between the independent variables (variance inflation factor [VIF] values were <10). Also variables were selected after checking their independence, where tolerance values were of >0.1. Homoscedasticity assumptions were checked using the Breusch-Pagan test, with a *p*-value of ≥of 0.05 indicating the absence of heteroscedasticity.

## Results

A total of 500 participants seeking infertility treatment for primary infertility were contacted by fertility and reproductive clinics. Among those 500 eligible ones 443 participants agreed to participate; response rate of 88.6%. Table 1 lists the sociodemographic details of the study participants. Approximately 71.1% were Jordanians with a mean age of 34±9.03 years. More than half of respondents were well educated with university degree or higher (52.4%), closely followed by high school qualification (36.1%), with a small percentage having no formal education (11.3%). Most of the respondents (63.2%) were working with an average total family monthly income of 594±454 Jordanian dinar and more than two thirds (77.9%) were medically insured. The majority of participants believe their health was excellent (34.1%) or at least very good (48.3%).

**Table 1:** - Socio-demographic characteristics of the study sample (N=443).

Variables	n (%)
***Hospital***	
JU	132 (29.8)
JUST	183 (41.3)
Occupied Palestinian territories’ centers	128 (28.9)
***Gender***	
Male	180 (40.6)
Female	263 (59.4)
***Weight in kg, mean±SD***	
Male	80.5±15.2
Female	68.7±12.4
***Smoking***	
Smoker	106 (23.9)
Non-smoker	296 (66.8)
Ex-smoker (more than 6 months)	23 (5.2)
Waterpipe	17 (3.8)
***Insurance type***	
MOH	225 (50.8)
Private	99 (22.4)
Others	32 (7.2)
***Suffering from chronic diseases (hypertension, diabetes, heart diseases, respiratory problems, and others)***	
Yes	59 (13.3)
No	386 (87.1)
***Suffering from Vitamin D deficiency***	
Yes	102 (23.0)
No	318 (71.8)

Values are presented as a number and precentage (%). Some data was missing, subsequently totals do not always add to 443.

JU: Jordan University, JUST: Jordan University of Science and Technology, MOH: Ministry of Health, SD: standard deviation

The infertility characteristics of study participants are presented in Table 2. The average duration of marriage was 7.13±6.10 years, while the mean duration of infertility was 6.69±6.78 years. Over 50% of the participants (54.0%) belonged to nuclear families. The cause of infertility was mainly reported to unknown cause (35.9%), the wife (31.6%), with 21.4% of cases being reported to be the husband, and 11.1% to the couple. There were different types of infertility treatment, such as IVF (36.6%), using only ovulation induction medications (23.0%), IUI (14%), or using more than one treatment method (21.7%). The average total cost of treatment since the problem had been diagnosed was 2936±4529 Jordanian dinar, and the average number of doctors who followed up the case were 2.8±2.6, with only 1.8% of participants asked for any psychological consultation for infertility-related stress.

**Table 2:** - Infertility characteristics of the study sample at baseline (N=443).

Variables	n (%)
***Family type***	
Nuclear	239 (54.0)
Extended	200 (45.1)
Period of seeking therapy for infertility in years, mean±SD	4.2±4.1
***Presence of any close relative who suffers from infertility***	
Yes	56 (12.6)
No	386 (87.1)
Number of IUI or IVF trials, mean±SD	1.5±1.0
Number of doctors who follow the case, mean±SD	2.8±2.6
Cost of infertility treatment per month in JD, mean±SD	190±395
***The ART cost is covered by the insurance***	
Yes	115 (25.9)
No	328 (74.0)
***Seeking any psychological consultation for the infertility related stress***	
Yes	8 (1.8)
No	304 (68.6)

Values are presented as a number and precentage (%). Some data was missing, subsequently totals do not always add to 443.

SD: standard deviation, IUI: intrauterine insemination, IVF: in vitro fertilisation, JD: Jordanian dinar, ART: assisted reproductive technologies

Independent T-tests results indicated that women (37.1±8.3) reported a higher need for parenthood score than men (34.8±7.4; *p*=0.005). On the other hand, and in terms of relationship concern, men (34.7±5.7) reported a higher score than women (32.1±5.9; *p*=0.008). In terms of global stress, social concern, sexual concern, and rejection of a child-free lifestyle, men and women displayed comparable trends in their ratings. Data are summarised in Table 3.

**Table 3:** - Summary of different infertility-related stress scores (N=443).

Infertility-related stress scores	Total couples	Male	Female	*P*-values
Global stress	152.4±21.5	150.6±20.7	153.7±22.1	0.14
***Areas of stress***				
Social concern	31.8±5.8	32.3±4.9	31.5±6.4	0.23
Sexual concern	27.1±6.5	27.1±6.1	27.1±6.8	0.92
Relationship concern	33.8±5.9	34.7±5.7	32.1±5.9	0.008*
Rejection of child-free lifestyle	25.7±6.6	25.1±6.6	26.1±6.5	0.117
Need for parenthood	36.1±8.0	34.8±7.4	37.1±8.3	0.005*

Values are presented as mean ± standard deviation (SD).

^*^ Significant at less than 0.05 level.

Simple linear regression was carried out to assess the predictive factors associated with the global stress score. All variables with a *p*-value of <0.05 in the simple linear regression analysis were entered into stepwise multiple linear regression to identify the significant and independent predictors of the global stress score. Table 4 shows that 6 variables, including country of origin, family type, presence of insurance, number of doctors who followed up the case, cause of infertility, and any relative suffering from infertility, were significant predictors of the global stress score (*p*<0.05).

**Table 4:** - Assessment of factors affecting global stress score among infertile couples (N=443).

Independent variables	Global stress scores			
	Simple linear regression		Stepwise multiple linear regression	
	Standardised Coefficients Beta	*P*-values	Standardised Coefficients Beta	*P*-values
Age	-0.030	0.584	-	-
Country (Jordan, occupied Palestinian territories)	0.394	<0.001*	0.244	<0.001*
Gender (male, female)	0.037	0.477	-	-
Smoking (smoker, non-smoker, ex-smoker, waterpipe)	0.081	0.12	-	-
Family type (nuclear, extended)	0.143	0.006*	0.123	0.035*
Education (illiterate, high school, BSc, or postgraduate)	-0.079	0.128	-	-
Working (yes, no)	0.039	0.460	-	-
Insurance (yes, no)	0.122	0.019*	0.083	0.161
Whole monthly salary	-0.097	0.138	-	-
Period of marriage in years	0.031	0.552	-	-
Period of being without children in years	0.085	0.102	-	-
ARTs (ovulation induction medication, IVF, IUI, or more than one type)	0.012	0.829	-	-
Number of ART trials	-0.128	0.108	-	-
Number of doctors who follow up the case	-0.133	0.011*	-0.155	0.013*
Cause of infertility (wife, husband, both wife and husband, or unknown cause)	-0.118	0.023*	-0.102	0.084
Cost of ART per month in JD	-0.045	0.474	-	-
Total cost of ART	0.108	0.07	-	-
Presence of any relative suffering from infertility (yes, no)	0.109	0.037*	0.101	0.081
Suffering from Vitamin D deficiency (yes, no)	0.088	0.099	-	-

^*^ Significant at less than 0.05 level. BSc: Bachelor of Sciences, ARTs: assisted reproductive technologies, IVF: in vitro fertilisation, IUI: intrauterine insemination, JD: Jordanian dinar

The global stress score was found to be significantly and independently correlated with 3 different variables (*p*<0.05), as shown in Table 4. Standardised beta values, which illustrate the separate contribution of each domain of infertility-related stress, revealed that country of origin (B=0.394; *p*<0.001) had the greatest contribution of unique variance, followed by family type (B=0.143; *p*=0.035), and that number of doctors who follow up the case (B= -0.133; *p*=0.013) contributed significantly to the model. The positive B values indicate that higher global stress scores were correlated with 2 factors in the final model; the country of origin and family type. But the negative B value of several doctors who followed up the case indicated that this variable in the final model was associated with a lower global stress score.

## Discussion

Infertile couples are substantially affected by issues of stress associated to infertility, such as sadness and anxiety. According to the study’s findings, women’s psychological health was significantly worse than men’s because of their desire to have children. Numerous international investigations revealed that the partner who has a reproductive issue seems to experience psychological anguish more frequently.^23,24^ Concerning the aetiology of infertility, this is confirmed in the current study where the female is more expected to be the cause of infertility than males. According to a recent meta-analysis, infertile women’s depression scores were substantially higher than those of fertile women.^9^ It has been demonstrated that depression among infertile Iranian males is far higher than it was among men in Western nations, particularly among smokers and people with poor levels of education.^25,26^

Palestinian participants, especially those who were living in an extended family type, have a higher perceived infertility global stress score compared to the Jordanian participants. This could be attributed to the persistent psychological pressure surrounding the political circumstances of oppression, lack of freedom, and many other occupational challenges.^27^ Infertility has a negative impact on women’s psychological well-being, according to studies carried out in the occupied Palestinian territories on the topic.^28,29^ It was found that infertile women experience greater psychological discomfort than fertile women.^28,29^ Only 1.8% of recruited infertile couples asked for any psychological consultation for infertility-related stress. This may be because many patients are unwilling to seek mental health services because of fear of stigmatization, lack of awareness of the value of psychological consultation, mistrust of the medical establishment, and higher costs associated with such consultation.^30^

A recent large meta-analysis showed that failed IVF trials increased the chance of negative emotions and stress among infertile spouses.^31^ While recurrent infertility treatments are frequently a reliable sign of stress and sadness brought on by infertility, infertility itself may not always be a good indicator.^32^ Our findings showed that the impact of several gynaecologists who followed up the cases through repeated infertility therapy trials on psychological health was significant in the case of infertility-related stress symptoms, possibly as a result of a greater sense of helplessness.

According to a recent large systematic review, the couples’ age, number of oocytes recovered, and embryo quality were considered the most critical predictors of infertility success.^33^ Even if there are numerous effective infertility therapies available, high costs could be a significant obstacle to finishing treatment cycles, especially in the absence of health insurance.^34^ Despite the increasing frequency of infertility, there are irregularities in the coverage, expenses, and reimbursement policies of health insurance.^35^ In fact, this may be the cause of the lack of therapy in underdeveloped nations.^36^ The average total cost of infertility therapy of the recruited couples was 2936±4529 Jordanian dinar; this cost is considered of concern in Jordan or occupied Palestinian territories with their limited resources. Comprehensive insurance requirements are linked to more ART use and multiple births per ART birth.^37^ The current study showed those participants who did not have insurance (21.9%) or the participants whose insurance did not cover the treatments costs (74.1%) suffer from ART costs and have higher perceived infertility global stress scores.

On the other hand, infertile couples’ mental health was not significantly impacted by their age, their level of education, the length and causes of their infertility, or the type of ART they had according to the results of the current study. Our results align with previous findings in other cultures worldwide.^24,38,39^ Infertile couples frequently experience tremendous isolation from the fertile world due to perceived or actual social rejection and a lack of understanding from relatives and friends regarding their extreme desperation.^30^ The current study showed that infertile couples living in an extended family type, especially those who do not have relatives suffering from infertility have higher global stress scores, have significantly higher psychological distress, and less evaluation of their general health than the nuclear family type. The extended family provided all members with either beneficial psychosocial support or unfavorable repercussions. While happy and exciting events were more frequent among couples from extended families who were not depressed, sad, and bereavement events were more frequent among couples from extensive families who were depressed.^40^ Studies found that couples from nuclear families had higher quality of life and health evaluation than did those in extended families; this could explain why the psychosocial health status of extended families members is affected more than the nuclear families.^40,41^

### Study limitations

Firstly, participants in the study were purposively selected which limits claims to typicality and generalisability. Hawthorne effect and over or under representation of measured outcomes is another limitation inherently associated with interview style data collection.

This study highlights the need for particular psychological therapies, which are currently lacking in public healthcare routines in both Jordan and Palestine, for infertile couples to manage potential mental health issues and achieve their reproductive aspirations. Future studies on the healthy lifestyle behaviours and quality of life from a larger number of couples undergoing different infertility are needed.

The results of this study have significant ramifications for practice, education, and research. First, the high rates of infertility-related stress found in this study highlight the need to increase awareness of this problem among health providers to facilitate its future identification, diagnosis, and management. Supportive counselling for infertile couples is highly recommended, particularly for couples of low levels of education level, low socioeconomic status, living in extended families, and suffering from prolonged infertility duration with a history of failed assisted reproductive treatment. The encouraging findings of this study serve as a springboard for additional, in-depth investigation, including interviews with selected couples, their immediate family members, and close relatives. The possibly circular association between stress related to infertility and diminished reproductive potential may be clarified through longitudinal research.

In conclusion, infertile couples have considerably worse psychological conditions than infertility-related stress symptoms and indications. Current study has demonstrated that being a woman is mostly responsible for the etiology of infertility. Only the need for parenthood has been impacted by gender; nevertheless, scores on other dimensions, such as overall stress, social anxiety, sexual anxiety, and rejection of a child-free lifestyle, were identical for both infertile males and females. In terms of the global stress score, it was strongly correlated with the infertility case’s country of origin, family type, and number of doctors. The psychological wellbeing of infertile partners is far worse than the stress-related infertility signs and symptoms. This study emphasizes the requirement for certain psychological therapies, which are presently absent from Jordanian and Palestinian public healthcare practices, in order for infertile couples to handle probable mental health concerns and achieve their reproductive aspirations.
